# Trans-catheter aortic valve implantation in a patient with membranous ventricular septal defect, sub-aortic band, and double-chambered right ventricle: a case report

**DOI:** 10.1093/ehjcr/ytaf063

**Published:** 2025-02-13

**Authors:** Hirotsugu Mitsuhashi, Takuma Yamasaki, Ryuta Nakamura, Yoshihide Uno, Imun Tei

**Affiliations:** Department of Cardiology, Ayase Heart Hospital, 2-16-7 Yanaka, Adachi-Ku, Tokyo 120-0006, Japan; Department of Cardiovascular Surgery, Ayase Heart Hospital, 2-16-7 Yanaka, Adachi-Ku, Tokyo 120-0006, Japan; Department of Cardiology, Ayase Heart Hospital, 2-16-7 Yanaka, Adachi-Ku, Tokyo 120-0006, Japan; Department of Cardiology, Ayase Heart Hospital, 2-16-7 Yanaka, Adachi-Ku, Tokyo 120-0006, Japan; Department of Cardiovascular Surgery, Ayase Heart Hospital, 2-16-7 Yanaka, Adachi-Ku, Tokyo 120-0006, Japan

**Keywords:** Trans-catheter aortic implantation, Aortic stenosis, Ventricular septal defect, Sub-aortic band, Double-chambered right ventricle, Case report

## Abstract

**Background:**

We report a rare case of trans-catheter aortic valve implantation (TAVI) in an elderly male with membranous ventricular septal defect (VSD), sub-aortic band, and severe aortic stenosis (AS). We discuss the safety and efficacy of the technique.

**Case summary:**

An 86-year-old male was admitted to our hospital with congestive heart failure due to low-flow low-gradient severe AS, a membranous VSD, a sub-aortic band, and a double-chambered right ventricle (RV). The patient was not deemed to be a surgical candidate because of advanced age and frailty even though surgical aortic valve replacement, VSD closure, sub-aortic band resection, and myectomy of RV would be considered as definitive treatment. Instead, we performed TAVI and VSD orifice closure using the skirt part of the self-expanding valve (26 mm Evolut Pro Plus™) because VSD occluder is not approved and thus not available in our country. The trans-catheter procedure resulted in a reduction of the mean aortic valve pressure gradient improved from 33 to 2 mmHg and a decrease in the shunt flow (Qp/Qs) from 1.9 to 1.2. The patient’s heart failure improved, and he was discharged to home 7 days after the procedure. He remained well and had not been admitted to hospital since discharge.

**Discussion:**

Trans-catheter aortic valve implantation using a valve skirt may be considered in a situation where a high-risk patient is inoperable and VSD closure devices are unavailable. To complete this procedure safely, meticulous pre-procedural evaluation and accurate positioning using trans-oesophageal echocardiogram are required.

**Learning points**

- To be aware of the potential alternative procedure for patients with aortic stenosis and ventricular septal defect.
- To know the importance of pre-procedural investigation and evaluation.
- To highlight the variation and combination of acquired and congenital heart diseases and their pathological impact/procedural risk.

## Introduction

There is no previous report to treat severe aortic stenosis (AS), membranous ventricular septal defect (VSD), and sub-aortic band only by using a single trans-catheter aortic valve (TAV), and its safety and efficacy in this condition is unknown. This procedure may be helpful in countries where VSD closure is unapproved.

## Summary figure

**Table:** 

December 2019	Percutaneous coronary intervention (PCI) for circumflex artery was performed in another hospital
February 2020	PCI for left anterior descending artery was performed in another hospital. Ventricular septal defect (VSD) was found, but no further test and treatment were performed as the patient was asymptomatic
5 April–29 April 2022	The patient was admitted to our hospital due to heart failure (HF). Low-flow and low-gradient type severe aortic stenosis and membranous VSD were found in echocardiogram. HF improved after diuretics and medical therapy and discharged home. Cardiac computed tomography and trans-oesophageal echocardiogram revealed a sub-aortic band and a double-chambered right ventricle in addition to severely calcified aortic valve and VSD. Coronary angiogram identified no significant stenosis, and the right heart catheterization showed pulmonary hypertension, pressure gradient inside of right ventricle (50 mmHg), and significantly elevated Qp/Qs (1.90)
28 May 2022	Trans-catheter aortic valve implantation was performed. The right heart catheterization showed Qp/Qs improved to 1.2. Pulmonary artery systolic pressure was improved from 53 to 25 mmHg
4 June 2022	He became asymptomatic and discharged home after rehabilitation
23 July 2022	He is independent in his daily life

## Case presentation

**Figure 1:**
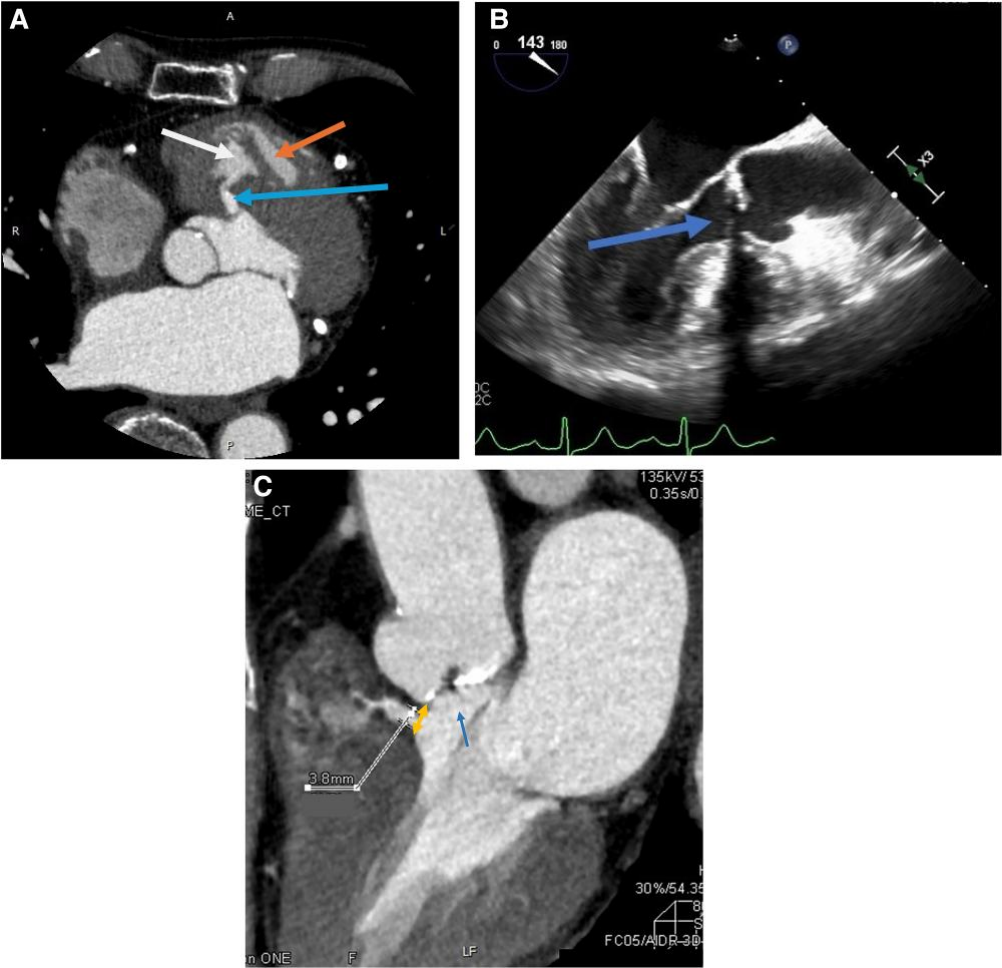
(*A*) Cardiac computed tomography revealed membranous ventricular septal defect and double-chambered right ventricle. Ventricular septal defect was connected to low-pressure chamber of right ventricle (blue arrow: ventricular septal defect, white arrow: low-pressure chamber, and orange arrow: high-pressure chamber). (*B*) Trans-oesophageal echocardiogram revealed a thin sub-aortic band (indicated by the blue arrow) attached to the left ventricular outflow tract wall just below the annulus level. (*C*) Cardiac computed tomography illustrating the longest distance between the annulus to the edge of the ventricular septal defect orifice was 7.3 mm (yellow arrow). The largest ventricular septal defect orifice diameter was 3.8 mm. Blue arrow indicating a fibrous and thin sub-aortic band.

An 86-year-old male, who had a previous history of hypertension and coronary artery disease had shortness of breath. He was admitted to our hospital due to heart failure (HF). He had undergone percutaneous coronary interventions in the circumflex artery in 2019 and the left anterior descending artery in 2020 both at another hospital. The patient was asymptomatic before admission. Blood pressure and heart rate were 176/91 mmHg and 85 b.p.m., respectively. Bilateral leg oedema was observed, with systolic murmur on auscultation. Chest X-ray revealed pleural effusion and congestion. Electrocardiogram (ECG) was within normal range. Blood test revealed elevated brain natriuretic peptide (150.4 pg/mL, normal range: <100 pg/mL). His STS and EuroSCORE 2 were 10.8 and 9.4, respectively. Trans-thoracic echocardiogram (TTE) revealed a left ventricular (LV) ejection fraction of 66% and a LV diastolic diameter of 41 mm. Calcified aortic valve was observed. The peak velocity of the aortic valve was 3.5 m/s, and the mean aortic valve pressure gradient (PG) was 33 mmHg. The aortic valve area (AVA) using the Doppler method was 0.78 cm^2^. The presence of a VSD can affect TTE-derived peak velocity and mean PG.^1^ Membranous VSD and double-chambered right ventricle (DCRV) were observed in cardiac computed tomography (CT) (*Figure 1A*). Trans-oesophageal echocardiogram (TOE), CT, and catheter-based AVA were 0.6–0.7 cm^2^. The calcium score was 2150 AgU. Based on these findings, we diagnosed his AS severe.

**Figure 2:**
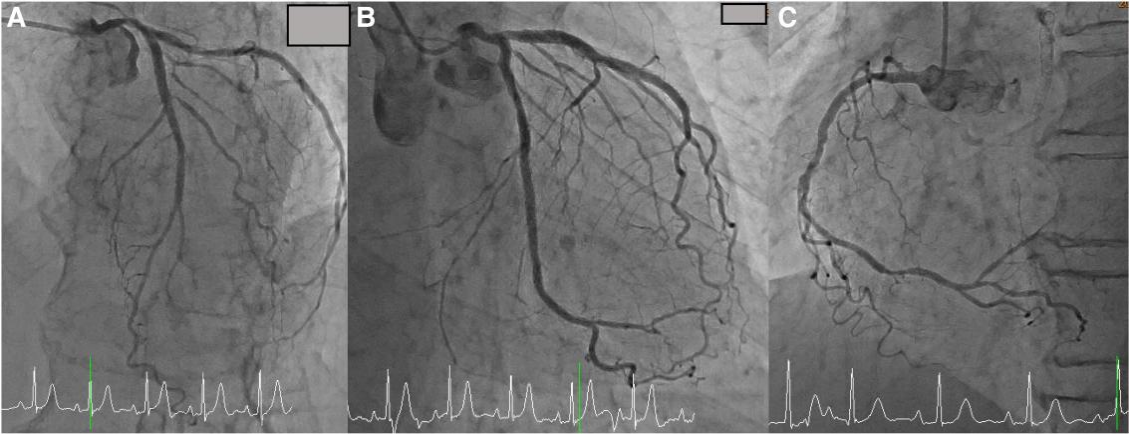
(*A*) Coronary angiogram showed no significant stenosis in the left anterior descending artery. (*B*) Coronary angiogram showed no significant stenosis in the circumflex artery. (*C*) Coronary angiogram showed no significant stenosis in the right coronary artery.

Cardiac CT showed his cardiac anatomy was suitable for TAV implantation (TAVI). Annulus perimeter, Valsalva diameter, and coronary heights of left coronary artery and right coronary artery were 66.1, 29.1, 14.6, and 18.7 mm, respectively. The VSD orifice was located 3.5–7.3 mm from the aortic valve annulus, and the orifice diameter was 3.8 mm. A thin sub-aortic band (thickness <1 mm) was attached to the LV outflow tract (LVOT) (*Figure 1B* and *C*). Coronary angiogram showed no significant stenosis and widely open stents (*Figure 2*). The catheter-based PG between LV apex and aorta was 27 mmHg. No PG was observed between LV apex and LVOT. The right heart catheterization showed normal wedge pressure (17 mmHg) and slightly decreased cardiac index (2.1 L/min/m^2^). Pulmonary hypertension (mean pressure: 38 mmHg) and a PG of 50 mmHg between high-pressure and low-pressure chamber of right ventricle (RV) were observed. The catheter-based Qp/Qs ratio was 1.9. We diagnosed severe AS in combination with left-to-right (L–R) shunt, due to VSD, as the leading cause of the patient’s HF. These findings fulfilled the indication for invasive treatment for AS and VSD based on the guideline.^2,3^

**Figure 3:**
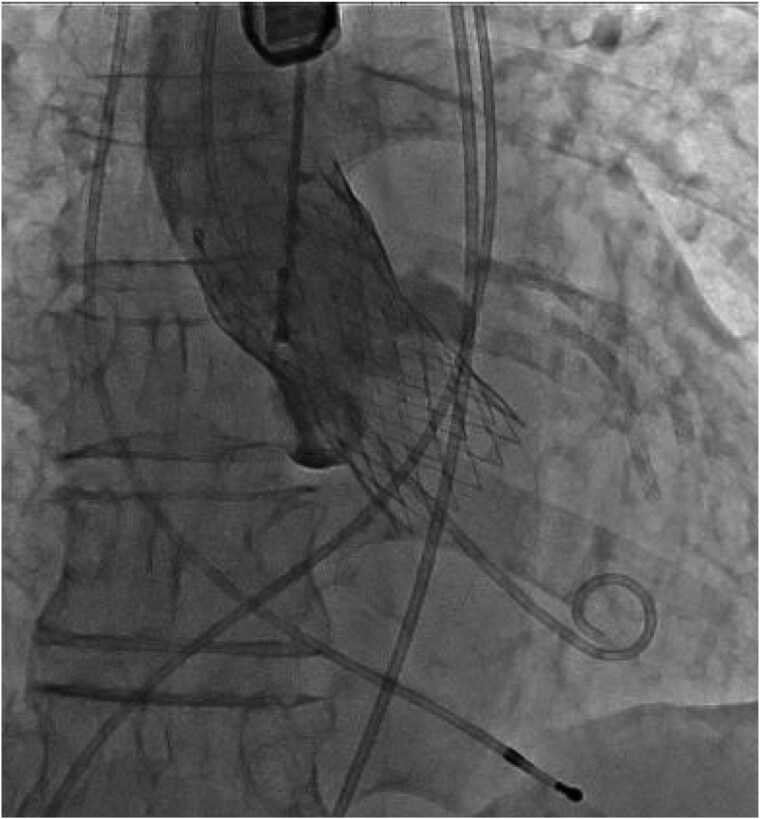
A 26-mm Evolut Pro Plus™ valve was implanted 9 mm below the annulus level.

**Figure 4:**
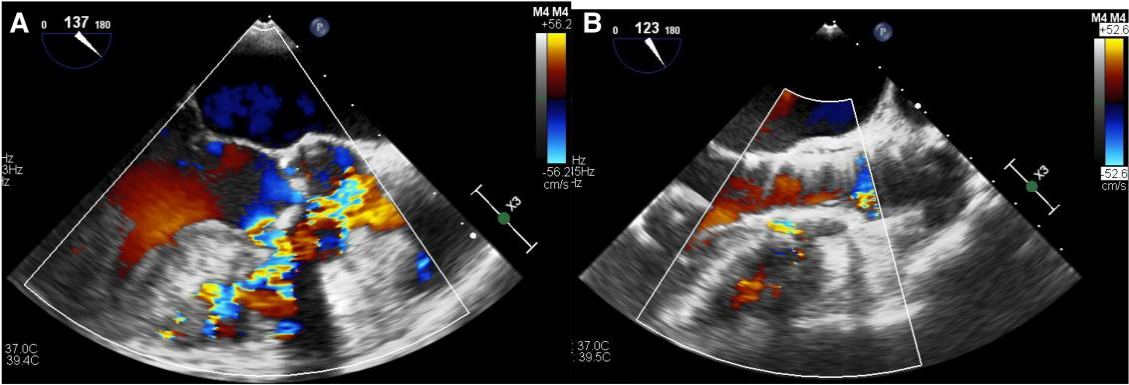
(*A*) Trans-oesophageal echocardiogram before trans-catheter aortic valve replacement revealed left-to-right shunt. (*B*) Trans-oesophageal echocardiogram after trans-catheter aortic valve replacement revealed the complete coverage of ventricular septal defect orifice by the valve and decreased left-to-right shunt without structural change of the left ventricular outflow tract and the left ventricle.

**Figure 5:**
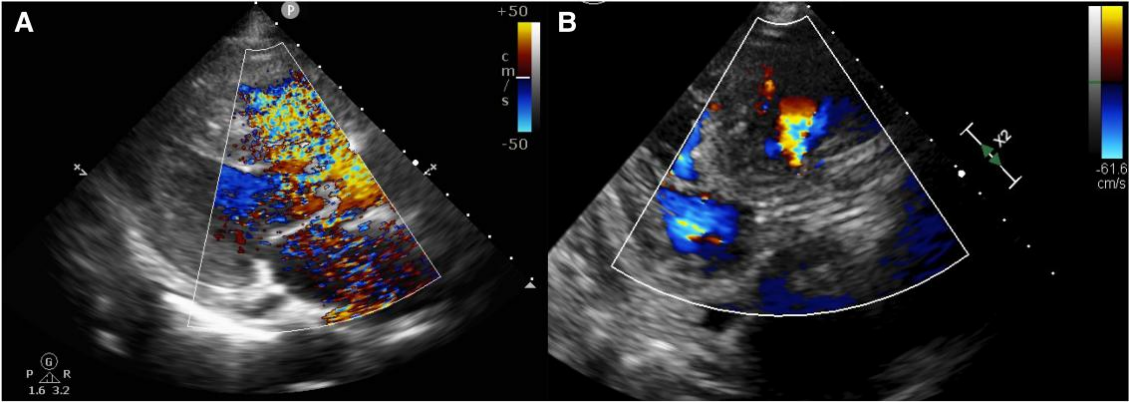
(*A*) Trans-thoracic echocardiogram before trans-catheter aortic valve replacement showing left-to-right shunt. (*B*) Trans-catheter aortic valve replacement showing decreased left-to-right shunt and good placement of the implanted valve.

Surgical aortic valve replacement, VSD closure, myectomy of the RV, and sub-aortic band resection treatment options were discussed with the heart team. The surgical team deemed not to be a surgical candidate because of the patient’s age, high risk of surgery and frailty (Clinical Frailty Scale: 5).^4^ As a result, a TAVI procedure with VSD orifice coverage using a valve skirt was planned as the interventional treatment option. We selected a 26-mm Evolut Pro Plus™ valve. Despite an increased risk of cardiac structural change due to pressing of sub-aortic band and/or injury of orifice of VSD and potentially deep implantation induced complete heart block, the valve could be deployed without complications. To treat AS and L–R shunt, the valve was selected because the valve has a long skirt at the bottom of the valve, which could cover the VSD orifice completely. Meanwhile, since the VSD orifice was located 3.5–7.3 mm below the annulus level, we deployed the valve 9 mm from the annulus level to cover the entire orifice of VSD with the valve skirt. We carefully deployed the valve by watching TOE to confirm no structural damage was made (*Figure 3*). The valve did not interfere with mitral valve movement. No leak across the valve and complete coverage of the VSD orifice with the valve skirt was observed by TOE. The amount of L–R shunt decreased (*Figure 4*). The catheter-based Qp/Qs ratio decreased from 1.9 to 1.2. Trans-thoracic echocardiogram–based aortic valve PG decreased from 33 to 2 mmHg. Post-procedural TOE revealed no leakage and a decrease in L–R shunt (*Figure 5*). The post-procedural ECG finding was unchanged (sinus rhythm, PR interval: 194 s and QRS duration: 70 ms). The patient was discharged from hospital 7 days after the procedure. He had not been admitted to hospital since discharge.

## Discussion

We were able to treat severe AS and to minimize the L–R shunt with a single device simultaneously with a simple TAVI procedure, and we could improve the patient’s condition clinically. Procedural risks included injury of VSD and an increased risk of pacemaker implantation due to a deep valve implantation. An additional long-term risk included endocarditis due to residual small L–R shunt.^5^ This technique should be considered on a case-by-case basis and particularly useful in high-risk patients for whom surgery is not feasible or in countries where a ventricular septal occluder (VSO) is not yet approved or unavailable. Unfortunately, VSO has not been approved in our country, and therefore, there was no option to implant a VSO before or after implanting the valve.

A DCRV is a congenital heart defect, in which the RV is separated into a proximal high-pressure and a distal low-pressure chamber. Double-chambered RV is frequently associated with a membranous VSD.^6^ In this case, co-existences of DCRV and VSD were observed, and it complicated diagnosis and treatment.

Iatrogenic VSD complications occur after TAVI,^7,8^ and the mechanism has been reported to be due to direct trauma to the septum from the implanted valve.^9^ Previous reports have indicated that iatrogenic VSDs are likely to occur with balloon expandable valves.^9^ Therefore, we selected a self-expanding valve.

In this case, the TAV needed to be implanted relatively deep in the LVOT. This deep implantation is a risk factor for post-procedural permanent pacemaker implantation.^10^ Other risk factors include complete bundle branch block and valve oversizing rate.^10^ The patient had no pre-procedural conduction defect, and the oversizing rate was only 10%. Consequently, there was no post-procedural conduction defect despite a deep valve implantation. Moreover, when a VSD closure device is used, post-procedural pacemaker implantation rate is reported as 5%.^11^ Thus, even if a shallow TAVI with a VSO implantation was performed in this case, the risk of post-procedural pacemaker implantation would be similar.

A sub-aortic band is also considered to be a congenital heart disease. It is usually thin, fibrous, or fibromuscular.^12^ The implanted valve may press down on the band, and it may cause LVOT obstruction. Hence, the safety and the efficacy of the procedure were assessed, and pre-procedural TOE and cardiac CT were very important in our decision-making. Trans-oesophageal echocardiogram during the procedure was also important to prevent LVOT obstruction and other complications. The location and size of the VSD orifice, and the position and thickness of the fibrous sub-aortic band fit well for a self-expanding valve in this case. Moreover, a self-expanding valve is retrievable and can be redeployed if the position is inaccurate or if structural changes take place.

## Data Availability

The data underlying this article will be shared at reasonable request to the corresponding author.

## Lead author biography

Dr Hirotsugu Mitsuhashi is an interventional cardiologist and a clinical scientist. His specialty in interventional cardiology includes PCI, TAVI, LAA closure, and ASD/PFO closure. He was selected as a global master of TAVI at the TCT 2022.
